# Tau drives translational selectivity by interacting with ribosomal proteins

**DOI:** 10.1007/s00401-019-01970-9

**Published:** 2019-02-13

**Authors:** Shon A. Koren, Matthew J. Hamm, Shelby E. Meier, Blaine E. Weiss, Grant K. Nation, Emad A. Chishti, Juan Pablo Arango, Jing Chen, Haining Zhu, Eric M. Blalock, Jose F. Abisambra

**Affiliations:** 1 Department of Neuroscience and Center for Translational Research in Neurodegenerative Disease, University of Florida, 1275 Center Drive, BOX 100159, Gainesville, FL 32610 USA; 2 Sanders Brown Center on Aging, Department of Physiology, Spinal Cord and Brain Injury Research Center, and Epilepsy Center, University of Kentucky, Lexington, KY 40513 USA; 3 Department of Molecular and Cellular Biochemistry, University of Kentucky, Lexington, KY 40513 USA; 4 Department of Pharmacology and Nutritional Sciences, University of Kentucky, Lexington, KY 40513 USA

**Keywords:** Tau, Transcriptome, Translation, Nascent proteomics, Ribosome, rpS6

## Abstract

There is a fundamental gap in understanding the consequences of tau–ribosome interactions. Tau oligomers and filaments hinder protein synthesis in vitro, and they associate strongly with ribosomes in vivo. Here, we investigated the consequences of tau interactions with ribosomes in transgenic mice, in cells, and in human brain tissues to identify tau as a direct modulator of ribosomal selectivity. First, we performed microarrays and nascent proteomics to measure changes in protein synthesis. Using regulatable rTg4510 tau transgenic mice, we determined that tau expression differentially shifts both the transcriptome and the nascent proteome, and that the synthesis of ribosomal proteins is reversibly dependent on tau levels. We further extended these results to human brains and found that tau pathologically interacts with ribosomal protein S6 (rpS6 or S6), a crucial regulator of translation. Consequently, protein synthesis under translational control of rpS6 was reduced under tauopathic conditions in Alzheimer’s disease brains. Our data establish tau as a driver of RNA translation selectivity. Moreover, since regulation of protein synthesis is critical for learning and memory, aberrant tau–ribosome interactions in disease could explain the linkage between tauopathies and cognitive impairment.

**Electronic supplementary material:**

The online version of this article (10.1007/s00401-019-01970-9) contains supplementary material, which is available to authorized users.

## Introduction

Neurons require constant protein production for synaptic function and are, therefore, particularly vulnerable to chronic attenuation of protein synthesis [35]. Transient suppression of translation is a cellular strategy to overcome conditions such as endoplasmic reticulum stress [20]. However, chronic suppression of protein synthesis contributes to the pathogenesis of multiple neurodegenerative disorders including tauopathies [4]. Pronounced ribosomal deficiencies appear in regions where tau pathology is evident, yet the link between tau and ribosomal function has not been established [14]. Furthermore, memory formation requires protein synthesis [15, 22]. Since progressive memory loss is a common and early symptom of virtually all tauopathies, and the processes of learning and memory are intricately dependent on de novo protein synthesis, ribosomal dysfunction could be an underlying mechanism driving these disorders.

Tau normally binds to ribosomes in the brain, and this interaction is enhanced in tauopathies [34]. In fact, hyperphosphorylated tau complexes with ribosomes in early stages of pathological tau aggregation [25, 38, 39, 42–44, 57], and tauopathic brains have reduced ribosomal function [14, 28, 32, 50]. These data suggest that alterations to the tau–ribosome complex could be an early pathogenic event in these disorders.

Our recent studies show that ribosomes associate with both pathological and non-pathological tau [35, 36]. Yet, the consequences of these interactions are still unknown. The emerging concept of ribosome specialization, where accessory proteins promote ribosomal selectivity for translation of distinct mRNAs, suggests an entirely new mechanism for regulation of protein synthesis [8, 53, 54, 61]. Considering that tau associates with ribosomal proteins [19] and that pathological tau modifies the rate of translation [34], we hypothesized that tau alters ribosome function thereby promoting translation of distinct transcripts.

To test this hypothesis, we used several in vivo and in vitro models, as well as human Alzheimer’s tissue, where disease-associated tau species are enriched. We show that tau expression impairs protein translation by measuring protein synthesis in vivo in the brain with puromycin labeling of nascent peptides. Then, using a transcriptomics-to-proteomics approach, we identified a tau-driven disparity between gene transcription and protein synthesis. Interestingly, tau decreased protein synthesis of ribosomal genes but not their transcription in tau transgenic mice. We hypothesized this unequal distribution was based on tau altering the function of ribosomal protein S6 (rpS6 or S6), which is involved in the regulation of ribosomal protein synthesis [18, 62]. We also found that tau interferes with S6 activation, and this interaction correlates with the decreased translation of transcripts coding for ribosomal proteins in Alzheimer’s disease (AD) brains. Consistent with our previous findings, these new data suggest the overall loss of translation found in tauopathies may be the result of a pathological gain-of-function of tau, where it attenuates translation by reducing the function or availability of S6.

## Methods

### Mice

The Institutional Animal Care and Use Committee (IACUC) of the University of Kentucky approved the use of animals in this study, which were conducted in accordance with the principles of animal care and experimentation in the Guide For the Care and Use of Laboratory Animals. Parental rTg4510 (Tg) mice were obtained from The Jackson Laboratories (stock #024854) and backcrossed for at least five generations onto FVB/NJ non-transgenic (Non) mice (stock #001800) and genotyped as described previously [51]. Tg mice and Non mice (littermate controls) were housed in a 14 h light/10 h dark cycle at a constant temperature (23 °C ± 2 °C) with food and water available ad libitum. Doxycycline treatment consisted of feeding mice a doxycycline diet (200 ppm, Envigo TD.00502) for 35 days with animals killed on the final day of treatment [4]. Tg and Non mice used for puromycin immunostaining were gavaged once a day for 30 days with 0.5% hydroxypropyl methylcellulose + 0.1% Tween-80 in water at pH 4.

### Human brain samples

Human samples were obtained from the University of Kentucky (UK) Alzheimer’s Disease Center. Sample collection and experimental procedures involving human tissue were in compliance with the UK Institutional Review board. Samples from Brodmann areas 21/22 (superior temporal gyrus) were used. Patient demographics are included as a table in Online Resource 1.

### In vivo puromycin administration

Mice were injected intraperitoneally with 225 mg/kg puromycin suspended in water (Research Products International, P33020). After 25 min, they were placed in an isoflurane anesthesia chamber for 5 min. Approximately 5 min following isoflurane exposure, animals were transcardially perfused for 5–10 min post injection with 0.9% saline. Brains were immediately harvested and the hemispheres anatomically separated and snap frozen in liquid nitrogen for downstream processing or drop-fixed in 4% para-formaldehyde for immunohistochemical studies. Brain laterality was maintained throughout experiments.

### Puromycin immunohistochemistry

Immunohistochemistry was performed as described previously [2]. Briefly, 4% para-formaldehyde drop-fixed brain samples were cryoprotected by incubating in sequential concentrations of sucrose (10%, 20%, and 30%) sucrose for 24 h each. Samples were frozen on a temperature-controlled freezing stage, sectioned (25 µm) on a sliding microtome, and stored in a solution of PBS containing 0.02% sodium azide at 4 °C. Free-floating tissue was treated with 3% (v/v) hydrogen peroxide + 10% (v/v) methanol in Tris-buffered saline (TBS, pH 7.4) to quench endogenous peroxidase activity. The Mouse on Mouse (MOM) Detection Kit (Vector Labs, BMK-2202) was used for blocking and staining procedures, with buffers prepared as described in standard protocol supplied with the kit. Sections were then incubated in Mouse Ig blocking buffer for 1 h at RT. Sections were incubated overnight at 4 °C with puromycin monoclonal antibody at 1:100 (EMD Millipore, MABE343) in MOM Diluent. Sections were washed with TBS and incubated with biotinylated anti-mouse IgG (Vector Laboratories) for 10 min at RT. Sections were washed again and incubated in ABC solution (Vector Laboratories) for 10 min at RT. Sections were washed again and incubated in diaminobenzidine (Sigma–Aldrich) and hydrogen peroxide in TBS. Sections were washed, mounted, and cover-slipped using Depex mounting media (Electron Microscopy Science). Images of the cortex (specifically somatosensory cortex, SSp) and hippocampus (CA1) were taken and quantified together for analysis of regions with severe tau pathology in rTg4510 mice. All values were normalized to signal in non-transgenic control mice.

### Sample tissue homogenization

Brain samples from human patients (~ 100 mg) or from mice (~ 50 mg) were mechanically homogenized in RIPA lysis buffer (Thermo 89900) with protease inhibitors (Sigma 4693159001), PMSF (1 mM final concentration), and phosphatase inhibitors (Gibco 786-452 and -451) as previously described [5]. Samples were centrifuged at 4 °C at 13,000×*g* for 15–25 min, and the supernatant was used for subsequent steps. Protein concentrations were quantified using the Pierce BCA kit (Thermo Fisher, 23225).

### Western blotting

Western blot experiments were performed as described previously [26]. Sample lysate protein concentrations were normalized with lysis buffer and denatured with 4 × Laemmli buffer with 10% β-mercaptoethanol. Proteins were resolved in 10% Tris–Glycine gels (BioRad) and transferred onto polyvinylidene fluoride (PVDF) membranes (Millipore, IPVH00010). Membranes were blocked in 1X PBS with 0.1% Tween-20 (PBS-T). All antibodies were diluted in 5% milk or 5% BSA in PBS-T. Primary antibodies were used as follows: PHF1 (1:2000, generously provided by Dr. Peter Davies), H150 total tau (1:2000, SantaCruz), Tau 5 total tau (1:2000, Millipore), actin (1:5000, Cell Signaling Technology), GAPDH (1:5000, Cell Signaling Technology), RPL28 (1:1000, GeneTex), EIF3E (1:1000, Sigma-Aldrich), Phospho-RPS6 Ser240/244 (1:1000, Cell Signaling Technology), total RPS6 (1:1000, SantaCruz). Bands were detected using ECL (GE Amersham Imager 600) using SuperSignal West Pico (Thermo Fisher, 1863096). Blot images were quantified using ImageJ (1.52b) and normalized to either GAPDH or β-actin.

### Puromycin immunoprecipitation

Exactly 400 µg of protein were brought to 500 µl with Hsiao-TBS and incubated with 5 µl of anti-puromycin antibody (Millipore, mabe434) overnight at 4° C under rotation. Approximately 150 µg Protein G Dynabeads (Thermo Fisher, 10003D) were resuspended in 50 µl 10 mM Tris (pH 7.5) and crosslinked with BS3 and then incubated with the sample-antibody complex for 2–3 h at RT under rotation. Beads were washed twice with washing buffer (10 mM Tris, 50 mM NaCl, pH 7.5) containing 0.2% Tween-20 and twice without Tween-20. Samples were eluted with 25 µl containing 100 mM glycine (pH 3.0) for 10 min at RT, and subsequently quenched with equal volume of 10 mM Tris (pH 8.0). Eluted sample protein concentrations were quantified at approximately 10 µg.

### Nascent protein proteomics

Proteins eluted from the puromycin immunoprecipitation were run via SDS-PAGE. Each lane in the gel was excised into 12 major portions and subjected to dithiothreitol reduction, iodoacetamide alkylation, and in-gel trypsin digestion using a standard protocol as previously reported [13, 63]. The resulting tryptic peptides were extracted, concentrated to 15 μl using a SpeedVac, and 5 μl were injected for nano-LC–MS/MS analysis [33]. LC–MS/MS data were acquired on an LTQ Velos Orbitrap mass spectrometer (Thermo Fisher Scientific, Waltham, MA) coupled to a Nano-LC Ultra/cHiPLC-nanoflex HPLC system (Eksigent, Dublin, CA) through a nano-electrospray ionization source. The tryptic peptide sample was injected with an autosampler, desalted on a trap column, and subsequently separated by reverse phase C18 column (75 mm i.d. × 150 mm) at a flow rate of 250 nL/min. The HPLC gradient was linear from 5 to 60% mobile phase B for 30 min using mobile phase A (H_2_O, 0.1% formic acid) and mobile B (90% acetonitrile, 0.1% formic acid). Eluted peptides were analyzed using data-dependent acquisition: peptide mass spectrometry data were obtained by Orbitrap with a resolution of 60,000. The seven most abundant peptides were subjected to collision-induced dissociation and MS/MS analysis in LTQ linear trap. The LC–MS/MS data were submitted to a local MASCOT server for MS/MS protein identification search via the ProteomeDiscoverer software. The mass error tolerance was 5 ppm for peptide MS and 0.8 Da for MS/MS. All peptides were required to have an ion score greater than 30 (*p* < 0.05). The false discovery rate in each LC–MS/MS analysis was set to be less than 1%. Two samples, one non-transgenic and one rTg4510, were immunoprecipitated with IgG antibody and analyzed via LC–MS/MS to determine inherent non-specific binding. Any matching proteins from these samples were removed from all samples prior to analysis. Only proteins with one or greater unique peptides were considered in the analysis. For pathway analysis, proteins which passed all filtering criteria were analyzed in the Database for Annotation, Visualization and Integrated Discovery (DAVID) [23] against the mouse genome. Only processes with an adjusted *p* value less than 0.1 were considered significant and selected for downstream analysis and are reported in Fig. 3. Since no unique annotations were reported between Non + Veh and Non + Doxycycline groups, the lists of proteins were combined to form the Non-group in analysis. Comparative ontology analysis was done by grouping significant annotation terms according to six distinct groups related to brain function. For each group, the number of terms were compared as a ratio to the number found in non-transgenic controls for the same class. Annotations were functionally grouped and presented as a ratio of the number of annotations assigned to each group relative to the number in Non + Veh. All proteins, brain-related annotation terms, and comparative analyses and grouping are reported in Online Resource 2.

### Microarray and transcript *post*-*hoc* template matching

Isolated RNA (100 μg per sample) was loaded onto a 96-well plate and shipped to Thermofisher (San Diego, CA) for array processing. High quality extracted RNA (RIN > 8.9) was labeled and hybridized to Mouse ClariomD microarrays (Clariom, ThermoFisher). One sample in the rTg4510 + normal feed (Tg + Veh) group did not pass quality control (PCA analysis and Pearson correlation matrix) and was removed from subsequent analyses. Signal intensities were calculated using the Robust Multi-Array Algorithm [11] and are reported on the log 2 scale. Transcript clusters were annotated to gene symbols using NetAffx annotation files (Release 36). The full transcriptional profile data set is available through the Gene Expression Omnibus under accession ID: GSE121264. For the purposes of this analysis, pre-statistical filtering was performed as in prior work [6, 16, 30], and included retaining uniquely annotated transcript clusters with reliable signal strength (RMA signal > 6.76 on at least 1 array). Intensity values of these pre-statistically filtered genes were then analyzed to identify differentially expressed genes (DEGs) with two-way ANOVA (*p* ≤ 0.01). For ANOVA analysis, the false discovery rate (FDR) estimate of multiple testing error is reported in Results. Although the ANOVA test identifies DEGs, it does not determine patterns of expression among those DEGs. To do this, DEGs were analyzed post hoc using a template matching strategy as in previous work [10, 16, 27]. Briefly, group mean intensities for each DEG were correlated with idealized templates representing six canonical expression patterns: rescued by tau reduction, resistant to tau suppression, altered by doxycycline treatment, altered by doxycycline and transgenic tau expression, altered by doxycycline only in non-transgenics, and altered by doxycycline only in transgenics. For example, the ideal template for “rescued by tau reduction” is represented by (0, 0, 1, 0) for Non + Veh, Non + Dox, Tg + Veh, and Tg + Dox groups. The mean intensities (log2) for example transcript Lyz1 are (5.64, 6.65, 7.29, 6.03), resulting in a correlation of *r *= 0.97 to this template. Finally, the sign of the correlation indicates whether the transcript matched the pattern (positive, e.g., upregulated) or matched the mirror-reflection of the pattern (negative, e.g., downregulated). DEGs were assigned to the template of highest correlation if Pearson’s *r* ≥ |0.85|. To further refine analysis, templates assigned significantly more genes than expected by chance (binomial test, *p* ≤ 0.05) were considered enriched and used for subsequent analysis. All DEGs and transcripts attributed to a pattern are listed in Online Resource 3.

### RNA isolation and quantitative real-time PCR

RNA was isolated from ~ 100 mg of human brain or ~ 50 mg of frozen cortex of rTg4510 and littermate control mice as described in [3] using TRIzol reagent (Ambion) with Proteinase K digestion (ThermoFisher, EO0491), then column purified using PureLink (ThermoFisher). RNA was measured for integrity on an Agilent 2100 Bioanalyzer and only samples > 8.9 RNA integrity number (RIN) were used in downstream analysis. RIN did not significantly differ between groups (*p* > 0.7). For qRT-PCR, gene transcription was evaluated by TaqMan probes and intensities were normalized to GAPDH expression as an internal control. Fold change was determined using the 2^−ΔΔ*Ct*^ method. Kruskal–Wallis and Dunn’s test *p* values are presented in Online Resource 4.

### Cell culture

We cultured tetracycline-inducible HEK cells which express wild-type human 0N4R tau, termed iHEK Tau cells, as previously described [4]. To induce tau expression, cells were treated with tetracycline (1 μg/ml; Sigma) for either 24 or 96 h (ON) and either immediately harvested or harvested following 24 h tetracycline washout (OFF) with fresh media. 60 min before harvesting across all groups, puromycin was added to the media to a final concentration of 10 μg/ml. Next, cells were washed 2× with ice-cold PBS and lysed using RIPA buffer (Thermo 89900) with protease and phosphatase inhibitors as previously described [34]. Protein concentrations were quantified using the Pierce BCA kit (Thermo Fisher, 23225).

## Results

We recently used in vitro models to demonstrate that misfolded, oligomeric, and hyperphosphorylated tau species reduce the rate of translation [34]. However, whether tau impairs translation in the brain remains unknown. Therefore, we measured changes in RNA translation using a puromycin-based assay (Surface Sensing of Translation or SUnSET) adapted for use in vivo [52]. As a structural analog of tRNA, puromycin is stably incorporated into growing polypeptide chains and these newly synthesized proteins can be detected with anti-puromycin antibodies. We performed SUnSET in 5mo and 7mo rTg4510 tau transgenic (Tg) mice to investigate whether tau alters protein synthesis as pathology increases. At 5mo, Tg mice have robust expression of a disease-associated human mutant tau (P301L), tau deposition into tangles, mild cognitive deficits, altered neuronal plasticity, and moderate brain atrophy. At 7mo, Tg mice have severe morphological and cognitive damage as well as extensive neuronal death and reduced protein synthesis [2, 47, 48, 51].

**Fig. 1:**
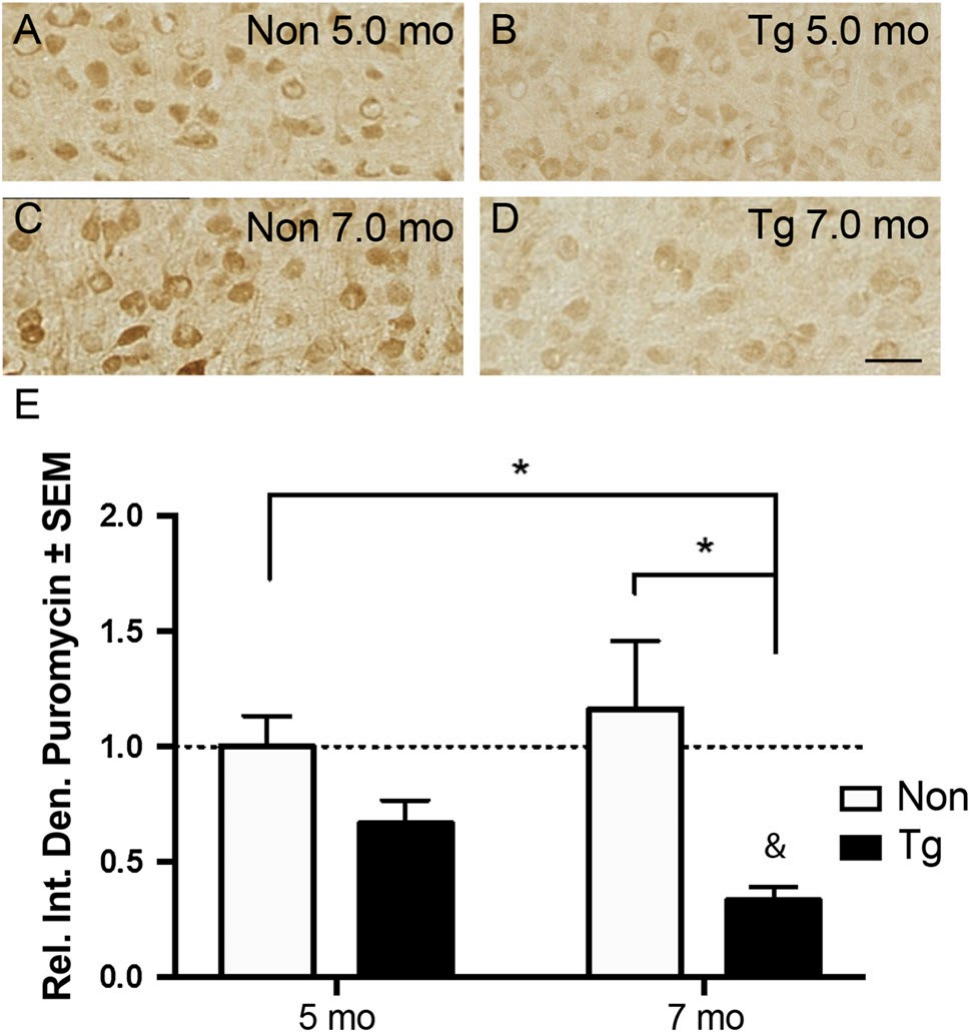
Protein synthesis is reduced in tau transgenic mice. Immunohistochemical staining of puromycin in non-transgenic (Non) and rTg4510 (Tg) at 5mo (**a**, **b**) and 7mo (**c**, **d**). **e** Quantification of panels **a**–**d** shows that puromycin signal was significantly reduced in 7mo Tg compared to control. Data analyzed by two-way ANOVA with Tukey’s multiple comparisons test. *p < 0.05 in comparisons outlined, & = 0.071 in comparison to 5mo Tg

We found that despite an appreciable but non-significant reduction at 5mo (~ 34%, *p* = 0.06), puromycinylated protein signal was significantly reduced in 7mo Tg mice compared to non-transgenic (Non) littermate controls in regions with strong tau expression (Fig. 1a–e). Interestingly, 7mo Tg mice also neared a significant reduction compared to 5mo Tg (~ 31%, *p* = 0.071). Global translation in the brain of Tg mice is impaired at 7mo but not at 4mo due to activation of the unfolded protein response mediated by the ER stress protein PERK [47]. To validate this previous report, we immunoblotted for p-eIF2α and total eIF2α levels in non-transgenic and Tg mice treated with and without a PERK inhibitor and detected no activation of the PERK pathway of the UPR at 5mo (Online Resource 5). However, since Tg mice have plasticity and cognitive deficits at 5mo [1, 2], we speculated that this time point may reflect tau-induced modifications in the types of proteins that are being synthesized rather than causing an overall decrease in the rates of protein synthesis.

**Fig. 2:**
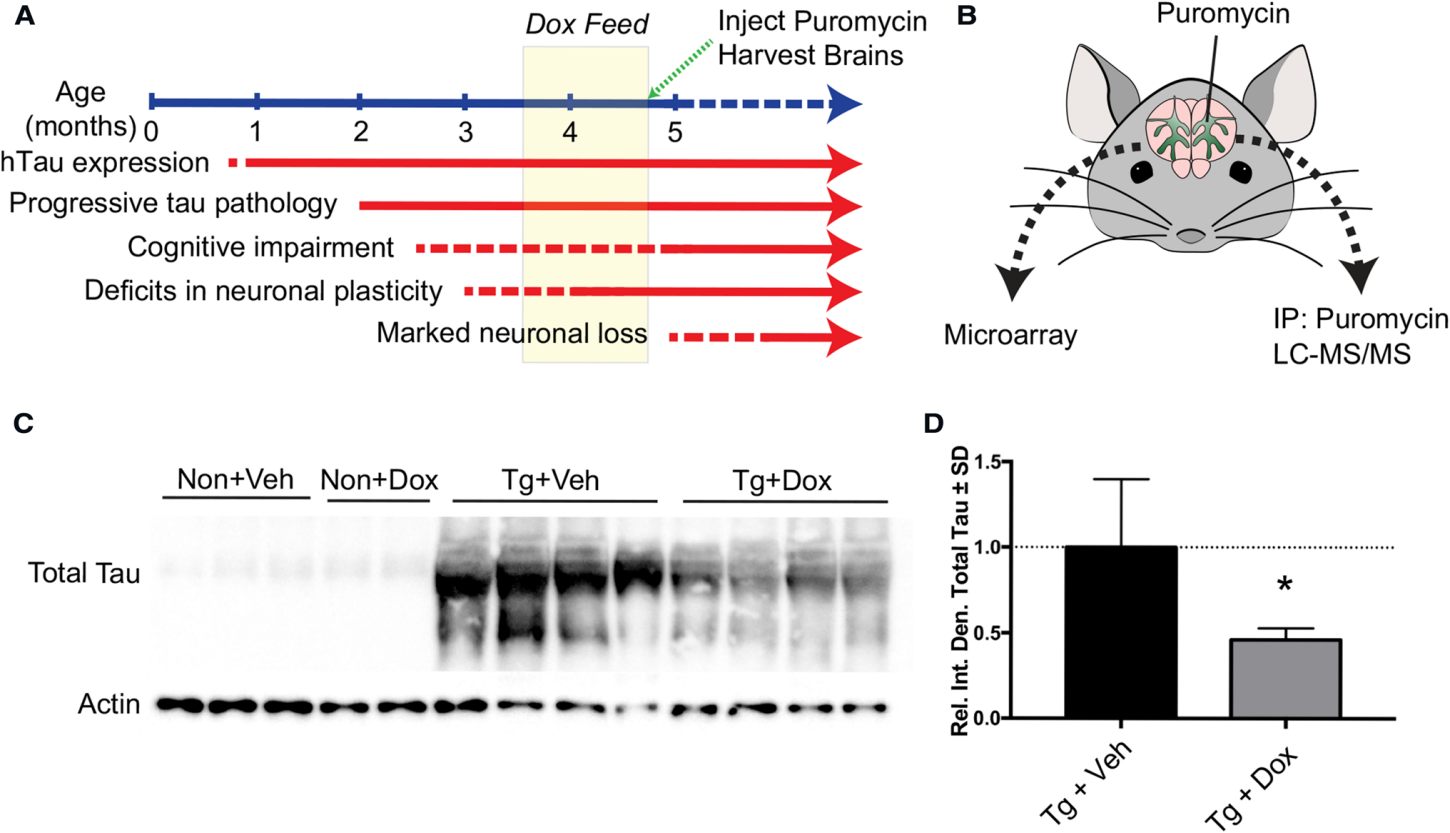
Experimental design. **a** Timeline of Tg phenotype and strategy for inhibiting tau expression with doxycycline (dox). **b** Strategy for brain isolation and processing for microarrays or puromycin-based proteomics. **c** Representative immunoblot showing reduced total tau (H150 antibody) levels after dox treatment compared to vehicle (veh) feed. **d** Quantification of **c** showing a 54% reduction in tau signal after doxycycline treatment analyzed by two-tailed, unpaired *t* test. *p < 0.05

To determine whether tau impairs the translation of select proteins, we suppressed tau expression in the TET/OFF Tg system featured in rTg4510 mice with a doxycycline diet for 5 weeks from 3.5 to 4.75mo (Fig. 2a). This paradigm of doxycycline treatment rescues cognitive dysfunction and other neuronal deficits [51]. Therefore, any changes detected in protein levels as a consequence of tau expression would identify proteins that participate in the earliest stages of the pathological process. Importantly, we could also rule out proteins that do not participate in cognitive alterations if their levels would not change. We coupled our in vivo SUnSET method with anti-puromycin immunoprecipitation to facilitate proteomic identification of newly synthesized proteins. By integrating this proteomic analysis with microarray measures of transcript levels (Fig. 2b), we sought to determine whether suppression of P301L tau expression rescues translation of select proteins. As expected and as previously reported, we confirm that the doxycycline treatment reduced tau levels by ~ 50% (Fig. 2c–d). This enabled the examination of the relationship between tau expression and RNA translation during the window of reversible cognitive dysfunction present in this model [52].

**Fig. 3:**
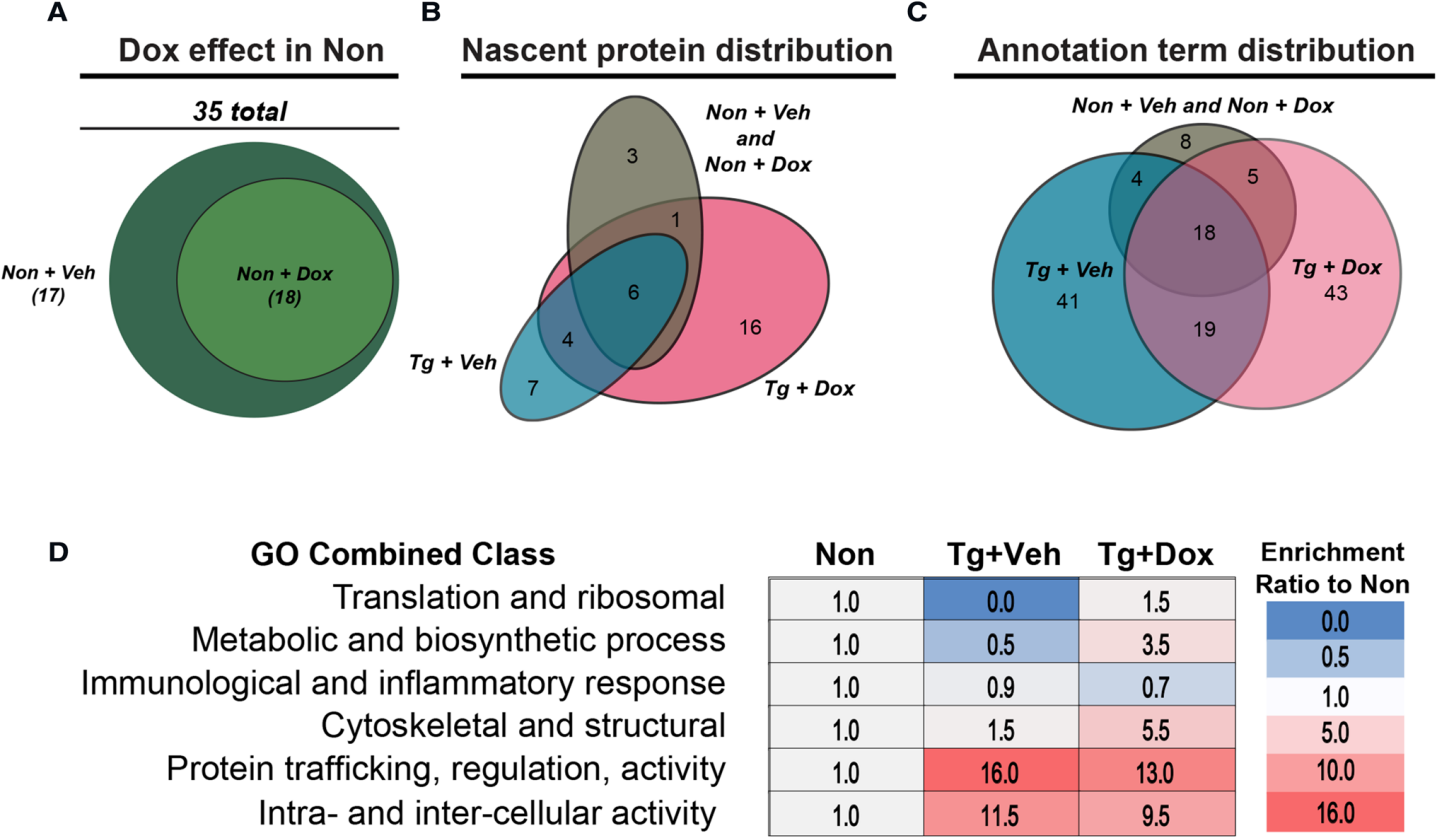
Pathological tau shifts the nascent proteomic profile. **a** Puromycinylated proteins were isolated by immunoprecipitation and analyzed using LC–MS/MS. **a** Doxycycline did not change the annotation profile in Non mice. **b** Venn diagram showing unique proteins identified by proteomics distributed between groups. **c** Venn diagram showing the distribution of annotation pathways representing proteins found in each group. **d** Categorized annotation terms identified in each group, separated by function and quantified as a ratio of the number of annotations present in Non. Ribosomal machinery and translation-related proteins were markedly reduced by tau expression and rescued with doxycycline treatment

To assess how the nascent proteome changed as a function of tau expression, we performed mass spectrometry analysis of nascent, puromycinylated proteins immunoprecipitated from cortical tissue of control and Tg mice treated with or without doxycycline feed. We first examined whether doxycycline treatment in non-transgenic mice had a functional effect on the cortical puromycinylated nascent proteome. Annotations identified by the Database for Annotation, Visualization and Integrated Discovery (DAVID) revealed no difference between Non mice with or without doxycycline treatment (Fig. 3a and Online Resource 2). This enabled the comparison between the nascent proteome of Tg mice with or without suppression of tau expression to non-transgenic mice. Strikingly, much of the puromycinylated nascent proteome varied due to tau expression and suppression (Fig. 3b). However, the total mass of puromycinylated proteins isolated from cortex was unchanged (Online Resource 6), validating the lack of statistically significant overall translation differences at this time point found by anti-puromycin immunostaining. Pathway analysis via DAVID of these protein groups showed similar variation in statistically significantly enriched annotation terms (Fig. 3c). We next heuristically grouped the annotations into six distinct categories to illustrate differences in the pathways represented by the puromycinylated nascent proteome when tau is overexpressed and then suppressed (Fig. 3d). As expected, the change in categories varied between tau expression and suppression. However, proteins involved in RNA translation and ribosomes were markedly reduced in tau-expressing mice, and doxycycline treatment rescued the synthesis of these proteins (Fig. 3d). These data indicate that tau expression reversibly altered the synthesis of translation machinery proteins in vivo, and it did so during the window where tau reduction rescues cognitive function.

**Fig. 4:**
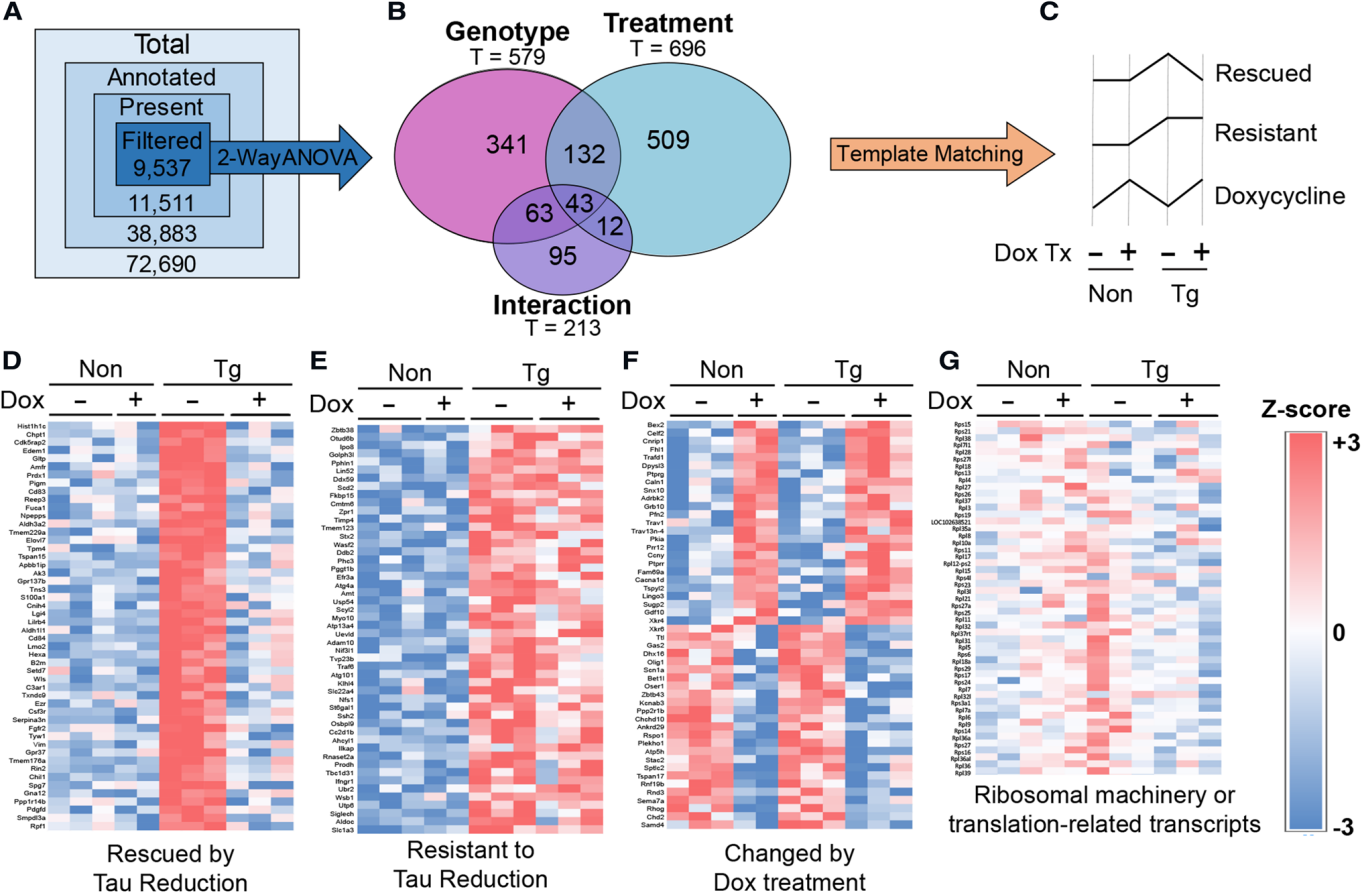
Pathological tau and doxycycline modify transcriptomic profiles into three distinct patterns, but ribosomal profiles do not change. **a** Filtering strategy of microarray results to identify genes and patterns of differential expression. **b** Venn diagram showing the distribution of transcripts identified as significant by two-way ANOVA (*p* ≤ 0.01) across effects of genotype, treatment, and the interaction between both variables. **c** Differential expression patterns enriched beyond the levels expected by random chance. **d**–**f** Heat maps representing intensities (log_2_) of transcripts matched to statistically significantly patterns of differential expression. **g** Heat map representing intensities (log_2_) of transcripts coding for proteins related to translation including ribosomal proteins.

To establish whether the changes in the translation of proteins identified from our proteomics approach were due to alterations in transcript levels, we profiled the transcriptome of Non and Tg mice treated with or without doxycycline using Clariom D microarrays. Differentially expressed genes (DEGs) were identified by two-way ANOVA (*p* ≤0.01; FDR 0.29; 1195/9537 genes—12.5%) and subsequently categorized by post hoc template matching (Methods: Fig. 4a–b; 91% of DEGs were assigned to one of these patterns). Three of the six templates were enriched above the levels of random chance and were considered as statistically significant patterns of differential expression (Fig. 4c, Online Resource 3). Pattern 1, “Rescued by Tau Reduction”, corresponded to 406 transcripts (~ 4%) rescued in transgenic mice given doxycycline (Fig. 4d). Pattern 2, “Resistant to Tau Reduction”, identified 106 (~ 1%) transcripts that increased in tau transgenic mice but unaffected by doxycycline treatment (Fig. 4e). Lastly, Pattern 3 established 333 transcripts (~ 3%) that were primarily affected by doxycycline treatment and not tau over-expression or suppression (Fig. 4f). Since this paradigm of doxycycline treatment rescues cognitive defects in Tg mice [51], these patterns suggest that we identified genes that mediate tau-driven cognitive impairment (Pattern 1), are not involved in cognitive rescue (Pattern 2), or that could be affected by doxycycline treatment paradigms (35d) used in this and other TET-dependent studies (Pattern 3). However, transcripts coding for proteins involved in translation were unchanged and were not categorized into any pattern of differential expression (Fig. 4g). Therefore, transcription was not a direct contributing factor to changes in the translation of ribosomal proteins, initiation factors, and other mediators of protein synthesis (Fig. 3d).

To define a mechanism by which tau exerts these changes, we focused on an innate process of translational regulation that is driven by the ribosomal protein S6. S6 is involved in regulating translation initiation and in facilitating the translation of ribosomal proteins, elongation factors, and initiation factors that contain a 5′ terminal oligopyrimidine (5′TOP) mRNA motif [24, 40, 49, 62]. These 5′TOP mRNAs are recognized by phosphorylated S6 to accommodate ribosomal engagement and subsequent translation [18, 37]. In addition, we previously identified that pathological tau associates with S6 in AD [34]. Therefore, tau-mediated impairment of S6 activity could at least partly explain why proteins involved in translation were reduced in Tg mice (Fig. 3d).

**Fig. 5:**
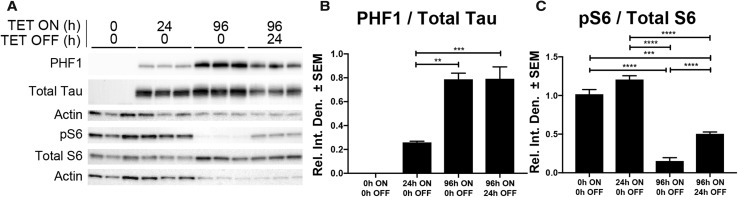
Tau expression reduces pS6 in immortalized human cells. **a** Representative immunoblot showing the effects of tau expression in tetracycline-inducible iHEK-Tau cells. Tau was expressed for 24 or 96 h (ON) and either harvested immediately or following 24 h tetracycline wash-out (OFF). Quantification of the ratio of PHF1 to total tau (H150 antibody) levels (**b**) or pS6 to total S6 levels (**c**). Data analyzed by two-way ANOVA with Tukey’s multiple comparisons test. **p < 0.01, ***p < 0.001, ****p < 0.0001

To investigate the impact of tau on S6, we modulated human wild-type 0N4R tau expression in iHEK tau cells and measured changes in active (pS6) and total S6 levels. iHEK-tau cells are a tetracycline-inducible cell line that stably expresses WT human tau [4]. As expected, tetracycline treatment progressively increased PHF1 and total tau levels compared to no tetracycline controls (Fig. 5a, b). These time points also correlate to protein synthesis impairments as measured by puromycin incorporation in this cell line as previously shown [36]. Compared to no-tetracycline controls the ratio of S6 phosphorylation to total protein was unchanged at 24 h of tau expression, but at 96 h the ratio was reduced by ~ 80% (Fig. 5c). Following 24 h tetracycline washout and subsequent decrease of tau level, pS6 to total S6 levels were rescued by 30% and approximately doubled the levels found at 96 h alone. This rescue of S6 phosphorylation suggested a potentially novel toxic gain-of-function where tau may preclude S6 phosphorylation.

**Fig. 6:**
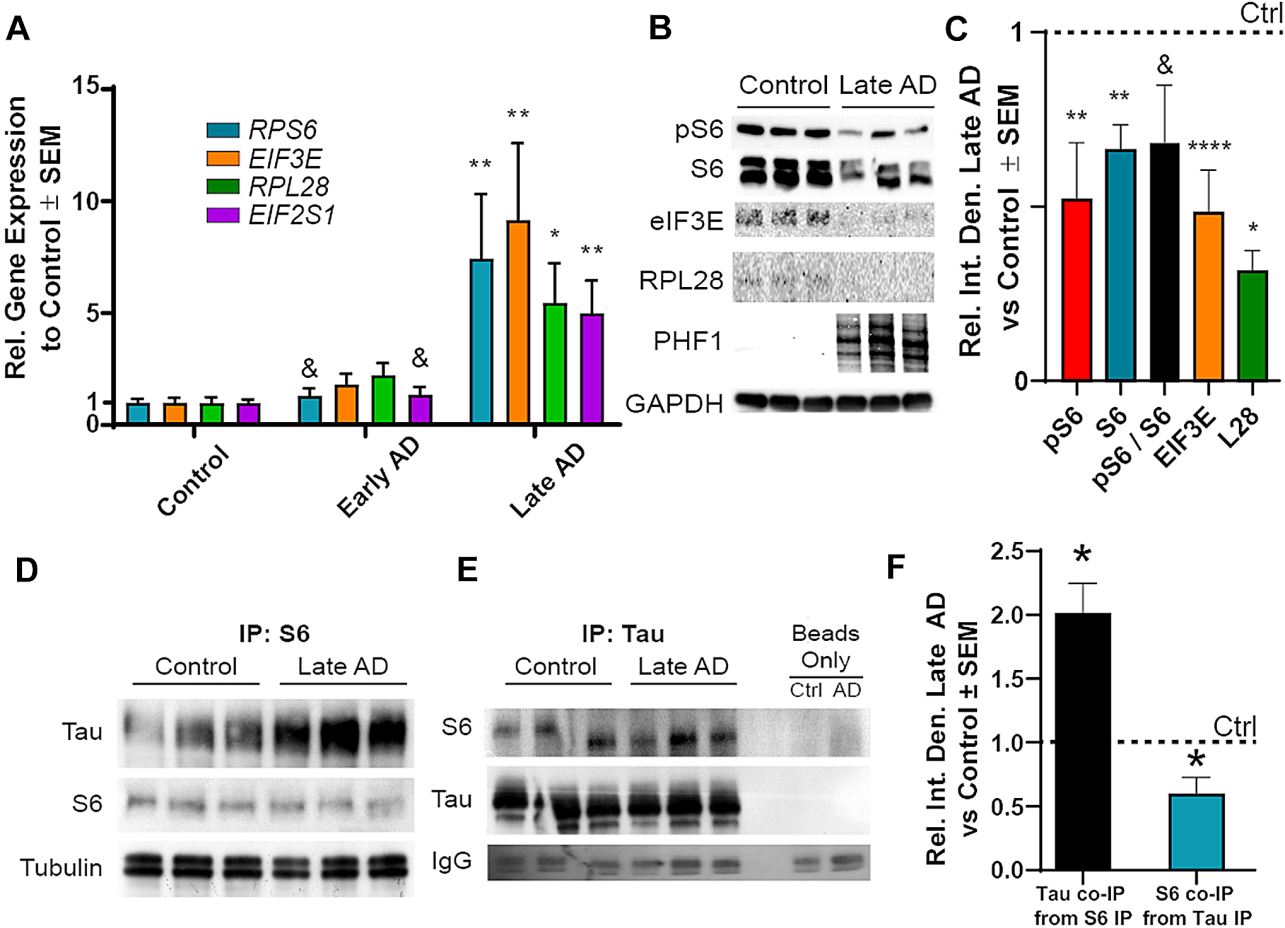
S6 exhibits impaired function and increased association with tau in AD. **a** RT-Q-PCR of human brain RNA isolate reveals no change in 5′TOP mRNA levels in early AD but substantial increases in late AD compared to control (Kruskal–Wallis with Dunn’s multiple comparison test, *n* = 9–10). **b** Representative immunoblot showing reduced pS6, total S6, and 5′TOP protein synthesis in late AD brains with PHF1 signal compared to control (one-way ANOVA with Tukey’s multiple comparison’s test, *n* = 6–12). **c** Quantification of pS6 and 5′TOP protein levels. S6 (**d**) and total tau (Tau 5 antibody, **e**) co-immunoprecipitate from human AD and control brain lysate regardless of host protein. Tissue lysate incubated without antibodies were used in the beads only lanes. IgG bands confirm the use of beads in all sample preparations, (*n* = 3). **f** Quantification of co-immunoprecipitation as a ratio to control. Each protein independently tested to control protein levels with two-tailed, unpaired Student’s *t* test. *p < 0.05, **p < 0.01, ***p < 0.001, ****p < 0.0001, ^&^*p* < 0.10

To establish whether these effects occurred during neurodegenerative processes, particularly conditions where tau is not overexpressed, we measured the RNA and protein levels of translation-related, 5′TOP mRNAs in human AD brains grouped by mini-mental state exam (MMSE) scores. While early AD (MMSE > 20–25) samples had no significant increases in relative transcript levels of four candidate 5′TOP transcripts (*RPS6*, *EIF3E*, *RPL28*, and *EIF2S1*) compared to control, late AD (MMSE ≤ 20) samples exhibited fivefold or greater increases relative to control in all candidate transcript levels (Fig. 6a). Two transcripts were also significantly increased in late AD samples compared to early AD (*RPS6* and *EIF2S1*). To assess whether these major differences in transcription evident in early and late AD brain samples, we compared changes in 5′TOP-coded protein levels in the corresponding early and late AD samples (Fig. 6b). The levels of active pS6 and total S6 were both markedly reduced (by approximately 50%; *p* < 0.01) in late AD samples compared to control, with the ratio of pS6/S6 trending toward a significant reduction (*p* = 0.092). Similarly, 5′TOP rpL28 and eIF3E were also significantly reduced (~ 55% at *p* < 0.001 and 75% at *p* < 0.05, respectively) (Fig. 6c) indicating that the protein synthesis of these translation-related transcripts was impaired as observed in tau transgenic mice (Fig. 3d).

Since pathological tau species associate with S6 in AD [33], and pS6 promotes translation of 5′TOP mRNAs [24], we speculated that AD brains contained tau–S6 complexes. We found that S6 and tau co-immunoprecipitated together in both control and late AD brains (Fig. 6d, e). Quantification of this signal showed a nearly twofold increase of tau associated with S6 in late AD relative to control, suggesting a potentially stronger interaction of S6 with disease-associated tau (Fig. 6f). Quantification of the S6 pool associated with tau revealed a 60% reduction in S6 compared to control, reflective of the decrease in total S6 found in late AD brain (Fig. 6b). These results show that tau–S6 complexes correlate with a reduced S6 function and consequently less ribosomal machinery in AD. Overall, these data suggest that tau associates with S6 and shifts the types of transcripts that are preferentially translated, eventually amounting to impairment in global translation.

## Discussion

Increasing evidence suggests that impaired translation contributes to the pathogenesis of neurodegenerative diseases like AD and ALS [29]. Over the last several decades, tau has been implicated as a modifier of a growing number of cellular processes across tauopathies, but the mechanisms of tau-mediated toxicity remain unclear [56]. Here, we expanded our previous findings on tau-mediated impairments in translation to show a potential mechanism describing how pathological tau modulates the selectivity and activity of ribosomes. We coupled transcriptomics and nascent proteomics with validation in vitro and in human brain samples from patients with AD to discern that tau reduces the activity of ribosomal protein S6, a crucial regulator of translation. In this context, we propose that tau pathology impacts translation, unveiling new prospects for therapeutic intervention. Since impaired translation may impact many pathways in tauopathies such as synaptic plasticity, cellular metabolism, and memory formation, tau-mediated impairments in translation may explain a mechanism by which tau directly promotes disease.

We establish that translation is shifted in early stages of progressive tau pathology in the rTg4510 mouse model of tauopathy (Fig. 1). We identified reduced protein synthesis at a later disease stage (7mo). Importantly, our in vivo SUnSET method validates a previous study that showed reduced ^35^S-methionine incorporation as a proxy of global translation at 7mo in rTg4510 [47]. Since no statistically significant decrease in protein synthesis has been determined by any study including our own at the cognitive-reversal window in rTg4510 mice, we hypothesized this earlier time point may reflect a turning point in translation that correlates with disease progression [51]. We, therefore, sought to couple transcriptomics and proteomic profiles of newly synthesized proteins, or nascent proteomics.

Parallel analyses of the transcriptome and nascent proteome revealed that, as expected, this early time point had marked differences in gene expression as a product of tau accumulation (Figs. 3, 4). As expected based on our previous results [34], synthesis of many proteins was suppressed by tau expression; however, we also identified many proteins that were increased as a consequence of tau expression, which corroborates results that were recently described [41]. Strikingly, transcript levels coding for ribosomal genes were unchanged while the protein levels were rescued by tau suppression, supporting the hypothesis that a shift in translation occurs during the window where doxycycline treatment rescues cognitive impairment in these mice. The results mirror past studies that found opposing effects on ribosomal gene transcript and protein levels in brain samples from patients with tauopathies [17, 28]. Moreover, these findings are not surprising considering the recruitment of tau–ribosome complexes in stress granules with TIA1 [7, 59] Based on our microarray analysis, we also isolated a pronounced doxycycline effect (Fig. 4f). Doxycycline treatment has been previously shown to alter physiological function and translation in vivo [36]. Interestingly, the affected genes corresponded primarily to inflammatory proteins. Since this potentially confounds the direct comparison of translation and multi-omics studies in our rTg4510 experiments, we sought to validate these findings in vitro. Importantly, to avoid potential issues with models of overexpression, we complemented our studies using human AD brain samples, which do not have tau overexpression [33].

**Fig. 7:**
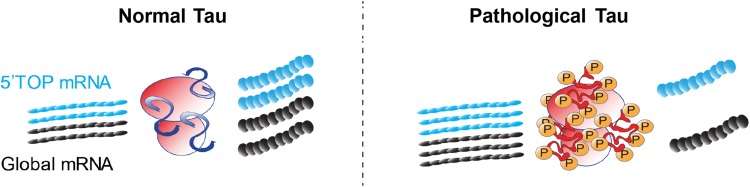
Schematic representation of overall conclusions. Tau normally associates with ribosomes and facilitates translation between global transcripts and 5′TOP mRNA sequences. In disease conditions, pathological tau species reduce the efficiency of translation and prevent rpS6 phosphorylation at S240/S244. As rpS6 activity decreases, translation of 5′TOP mRNAs coding for ribosomal and translational machinery is reduced, impairing global translation

While the reduction in total polysome levels [31], ribosome translational efficiency [17], and 5′TOP protein levels [17] have been well established in AD, no study has presented evidence mechanistically linking decreases in ribosome function with tau. We previously found that toxic tau species co-localized with S6 in AD brain [34], supporting other findings that tau associates with ribosomes [28, 40, 41, 43–45, 58]. Since S6 is a critical regulator of ribosomal biogenesis and activity, we investigated how the activity of S6 changes in tauopathic conditions in vitro and in human brains. We measured changes in S6 phosphorylation at Ser240/244 because this site is a marker for its localization into the somatodendritic domain, which is also where tau mislocalizes in disease [9, 18, 45, 46, 55]. The levels of pS6 were reversibly affected by tau levels in vitro (Fig. 5) and inversely proportional to PHF1 tau in AD brains (Fig. 6). This reduction in the functional pool of S6 also correlated with a fivefold or greater increase in transcript levels but a halving of protein levels of 5′TOP translation-related genes suggesting an impairment in 5′TOP transcript translation. We also identified a twofold increase in the amount of tau associated with S6 in late AD brains, suggesting that this decrease in S6 function is related to the direct association with tau in humans. Moreover, since reductions in functional pools of S6 correlate to reductions in LTP [48] and substantial dendritic atrophy [56], the tau–S6 complex may be pathologically relevant in the development or progression of cognitive dysfunction and merits further investigation. Overall, these data strongly suggest a gain of toxic function where tau associates with and prevents the phosphorylation of S6, thereby altering the selectivity and translational capability of the ribosome (Fig. 7).

While we present a mechanistic link between ribosomal dysfunction and tau pathology in AD, other potential direct and indirect factors may contribute to impair translation. For example, oxidative damage is linked to decreased ribosomal integrity and protein translation rates [21, 60], and the effect of tau on increasing oxidative stress is well established. Furthermore, toxic amyloid beta oligomers also induce rRNA damage and translational impairment [31]. Importantly, many other factors implicated in the pathogenesis of tauopathies alter S6 activity, such as S6K1 and S6K2. Interestingly, one study linked S6 kinase activity to AD phenotype in 5xFAD mice, where reducing S6 kinase activity conferred cognitive benefits [12]. This apparent discrepancy with our experiments may be due to the ability for S6 kinases to phosphorylate both active sites on S6 [9], which in turn have different and inconclusively determined effects in regulating S6 function and translation. Moreover, translation of 5′TOP transcripts is independent to S6K1 activity, suggesting that more potential factors regulate the function of S6 to regulate ribosomal function [58]. Ultimately, more sensitive measures to assess active translation are needed to more thoroughly elucidate the direct and indirect methods by which tau mediates translational impairments in disease.

## Electronic supplementary material

Below is the link to the electronic supplementary material.

Supplementary material 1. Human patient demographics. Clinical information regarding patient reference ID at the UK ADC, AD categorization, Braak stage, MMSE, age, sex, post-mortem interval, and brain weight (g). (TIFF 6109 kb)

Supplementary material 2. Puromycin-IP proteomic proteins and annotation terms. Proteins and annotation terms identified by nascent proteomics of puromycinylated protein immunoprecipitation from non-transgenic and rTg4510 mice treated with and without doxycycline. (XLSX 37 kb)

Supplementary material 3. Differentially expressed gene and pattern analyses assessed by Clariom D microarrays. Differentially expressed genes and statistically enriched patterns from microarray transcriptomics on non-transgenic and rTg4510 mice treated with and without doxycycline. (XLSX 412 kb)

Supplementary material 4. Q-RT-PCR statistical comparisons of 5′TOP mRNA transcripts in human AD brain samples. Kruskal–Wallis and Dunn’s multiple comparison test *p* values from Q-RT-PCR on human control and AD brain RNA isolate used in Fig. 6a. (TIFF 2224 kb)

Supplementary material 5. Phospho-EIF2α is not detectable at 5mo in rTg4510. 5-month old non-transgenic or rTg4510 tau transgenic mice treated with vehicle (0.5% hydroxypropylmethylcellulose + 0.1% Tween-80 in water at pH 4) or GSK2606414 (414), a PERK inhibitor, were harvested with RIPA lysis buffer. Cortical protein isolate was normalized and run on SDS-PAGE gel with a nine-monthold rTg4510 sample, an age previously reported to have UPR activity. Immunoblots probed for phospho-EIF2α (Ser51) or total EIF2α, with actinin as loading control (all from CST). No signal was found at the correct molecular weight for phosphor-EIF2α (~ 38 kDa) in 5mo mice. (TIFF 7972 kb)

Supplementary material 6. Puromycinylated protein quantity is unchanged due to transgenic tau expression or doxycycline treatment. Cortical protein lysate was isolated from 4-month and 3-week-old non-transgenic (Non) or rTg4510 tau transgenic (Tg) mice that were fed either normal (veh) or doxycycline (dox) feed for 5 weeks. Lysates were immunoprecipitated with puromycin (Millipore, mabe343) as described in Methods. The quantity of eluted puromycinylated proteins were assessed via bicinchoninic acid (BCA) assay and normalized to Non + Veh mice. (TIFF 8941 kb)
