# Supplementary figures and images for: Tau drives translational selectivity by interacting with ribosomal proteins
Source: Acta Neuropathol. 2019 Feb 13;137(4):571–83. doi: 10.1007/s00401-019-01970-9 (PMC6426815; doi:10.1007/s00401-019-01970-9)

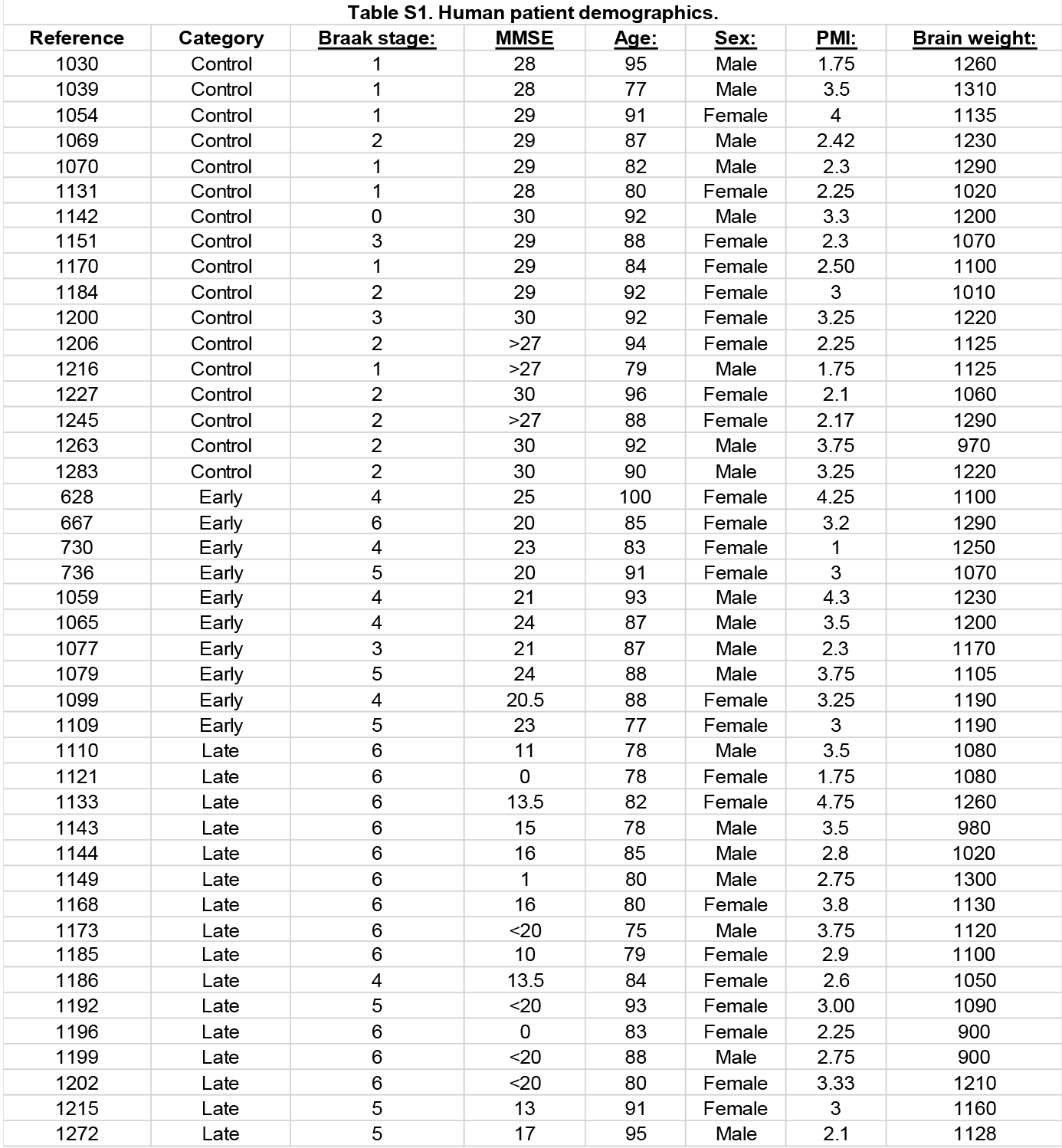

Supplement: Supplementary file 1 — Supplementary material 1. Human patient demographics. Clinical information regarding patient reference ID at the UK ADC, AD categorization, Braak stage, MMSE, age, sex, post-mortem interval, and brain weight (g). (TIFF 6109 kb) [file 401_2019_1970_MOESM1_ESM.tif]

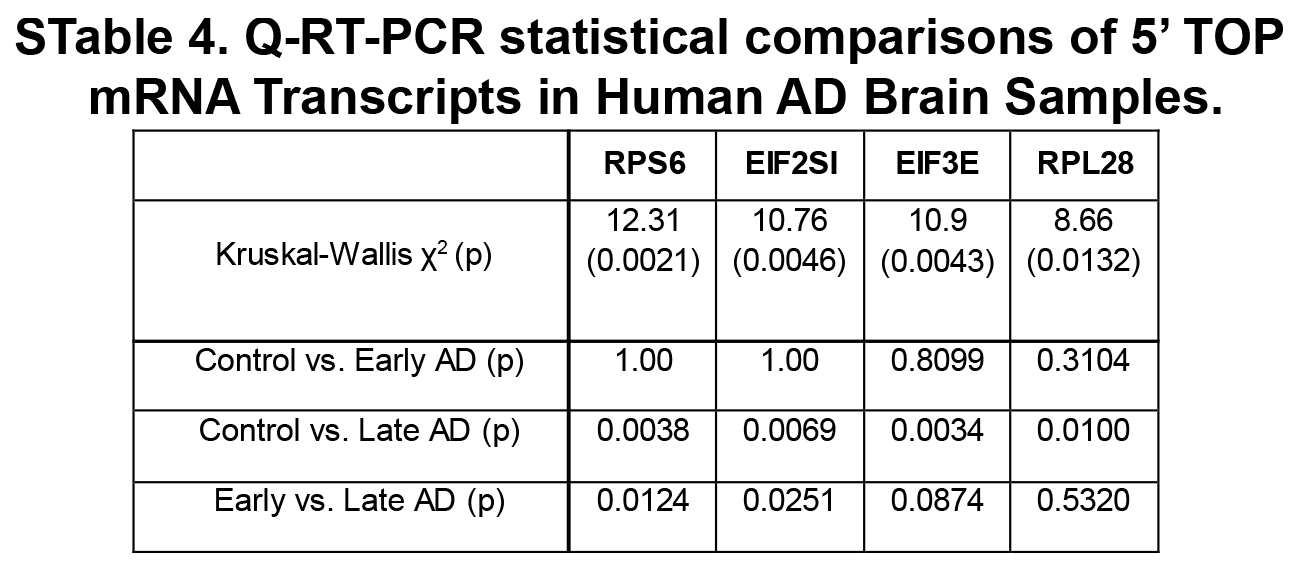

Supplement: Supplementary file 4 — Supplementary material 4. Q-RT-PCR statistical comparisons of 5′TOP mRNA transcripts in human AD brain samples. Kruskal–Wallis and Dunn’s multiple comparison test p values from Q-RT-PCR on human control and AD brain RNA isolate used in Fig. 6a. (TIFF 2224 kb) [file 401_2019_1970_MOESM4_ESM.tif]

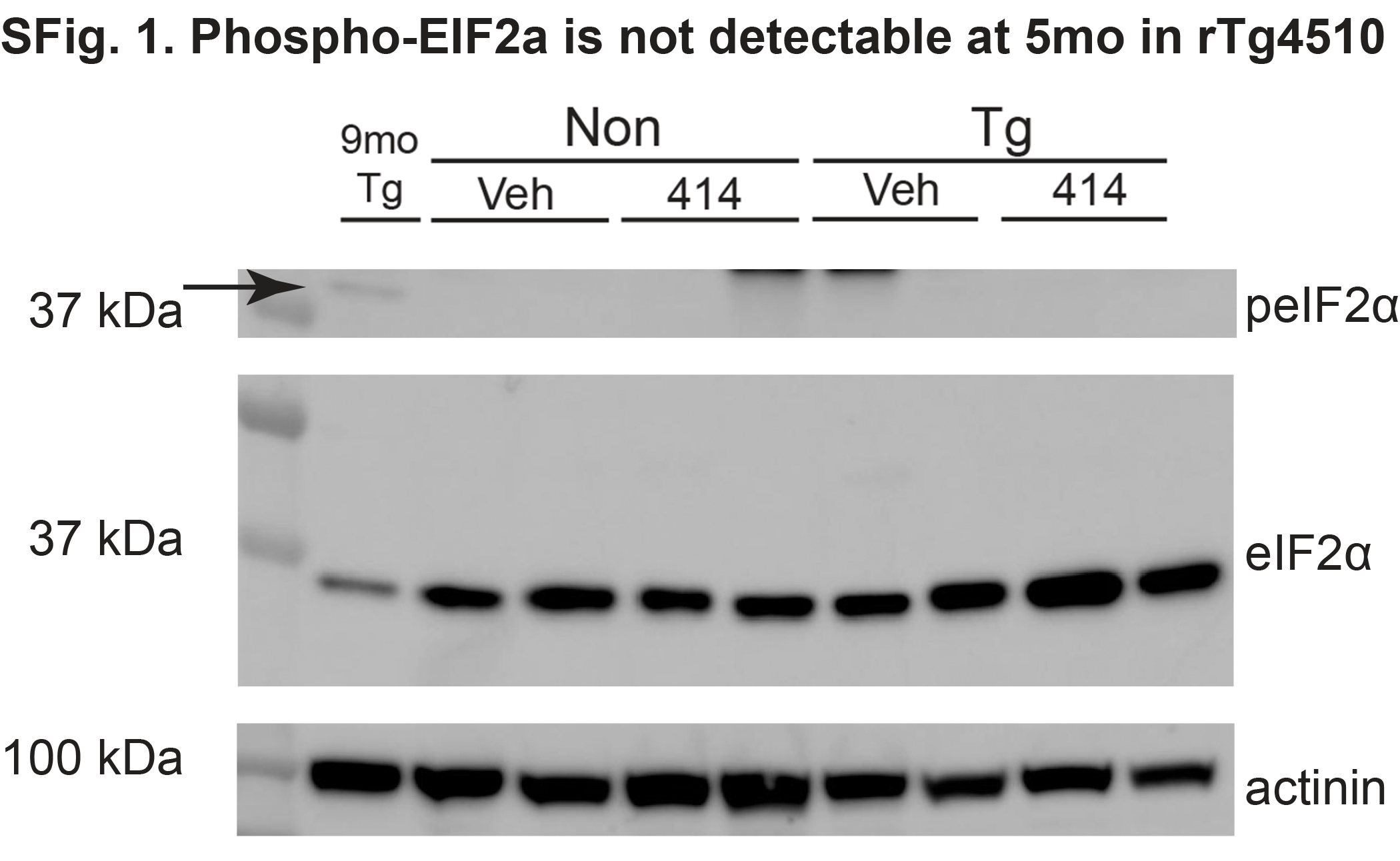

Supplement: Supplementary file 5 — Supplementary material 5. Phospho-EIF2α is not detectable at 5mo in rTg4510. 5-month old non-transgenic or rTg4510 tau transgenic mice treated with vehicle (0.5% hydroxypropylmethylcellulose + 0.1% Tween-80 in water at pH 4) or GSK2606414 (414), a PERK inhibitor, were harvested with RIPA lysis buffer. Cortical protein isolate was normalized and run on SDS-PAGE gel with a nine-monthold rTg4510 sample, an age previously reported to have UPR activity. Immunoblots probed for phospho-EIF2α (Ser51) or total EIF2α, with actinin as loading control (all from CST). No signal was found at the correct molecular weight for phosphor-EIF2α (~ 38 kDa) in 5mo mice. (TIFF 7972 kb) [file 401_2019_1970_MOESM5_ESM.tif]

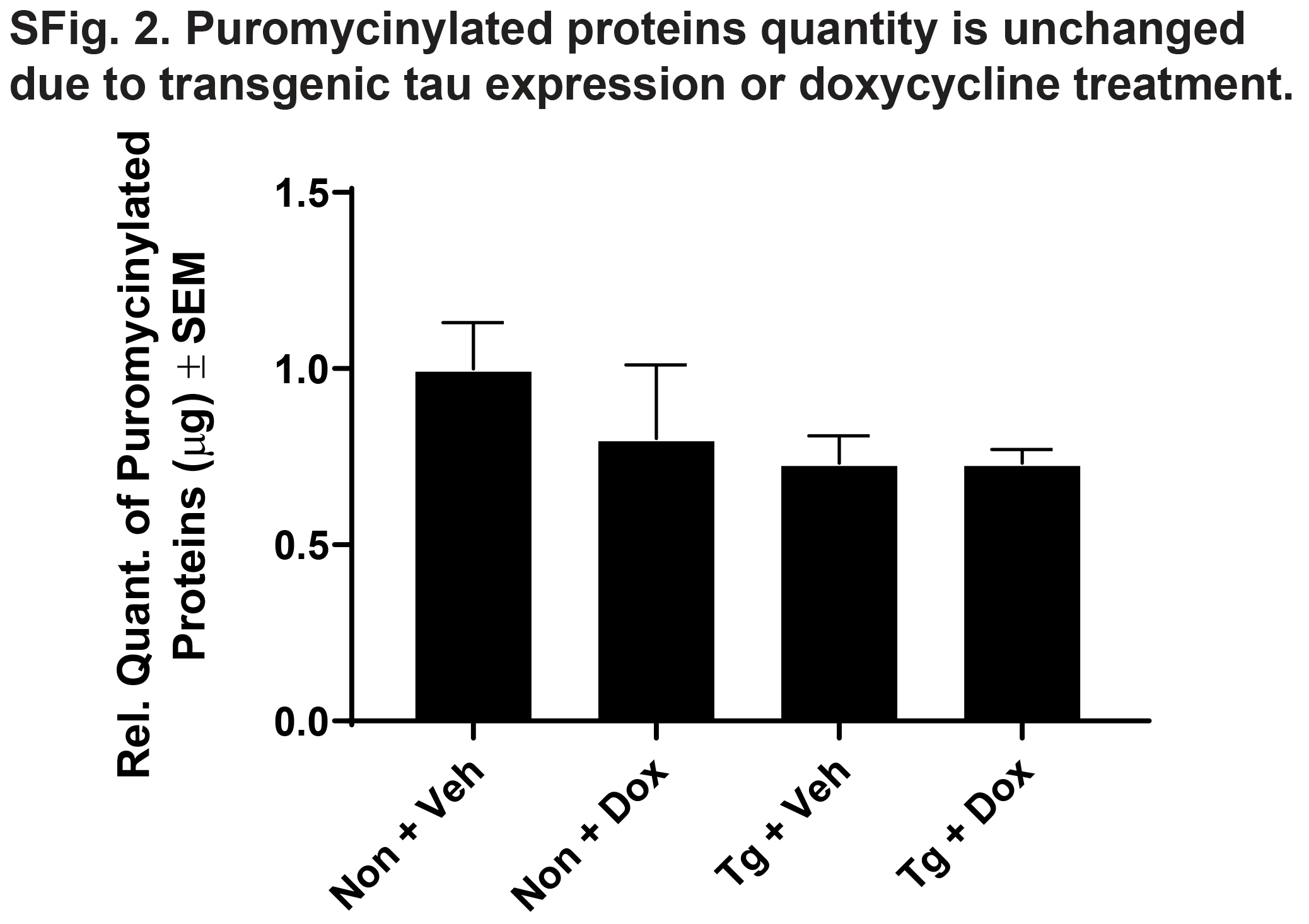

Supplement: Supplementary file 6 — Supplementary material 6. Puromycinylated protein quantity is unchanged due to transgenic tau expression or doxycycline treatment. Cortical protein lysate was isolated from 4-month and 3-week-old non-transgenic (Non) or rTg4510 tau transgenic (Tg) mice that were fed either normal (veh) or doxycycline (dox) feed for 5 weeks. Lysates were immunoprecipitated with puromycin (Millipore, mabe343) as described in Methods. The quantity of eluted puromycinylated proteins were assessed via bicinchoninic acid (BCA) assay and normalized to Non + Veh mice. (TIFF 8941 kb) [file 401_2019_1970_MOESM6_ESM.tif]
